# Stability of single skyrmionic bits

**DOI:** 10.1038/ncomms9455

**Published:** 2015-10-14

**Authors:** J. Hagemeister, N. Romming, K. von Bergmann, E. Y. Vedmedenko, R. Wiesendanger

**Affiliations:** 1 Department of Physics, Institute of Applied Physics and Interdisciplinary Nanoscience Center Hamburg, University of Hamburg, Jungiusstrasse 11, D-20355 Hamburg, Germany

## Abstract

The switching between topologically distinct skyrmionic and ferromagnetic states has been proposed as a bit operation for information storage. While long lifetimes of the bits are required for data storage devices, the lifetimes of skyrmions have not been addressed so far. Here we show by means of atomistic Monte Carlo simulations that the field-dependent mean lifetimes of the skyrmionic and ferromagnetic states have a high asymmetry with respect to the critical magnetic field, at which these lifetimes are identical. According to our calculations, the main reason for the enhanced stability of skyrmions is a different field dependence of skyrmionic and ferromagnetic activation energies and a lower attempt frequency of skyrmions rather than the height of energy barriers. We use this knowledge to propose a procedure for the determination of effective material parameters and the quantification of the Monte Carlo timescale from the comparison of theoretical and experimental data.

## Abstract (web summary)

Magnetic skyrmions are swirling magnetization textures which are topologically stabilized in helical magnets under an applied magnetic field. Here, the authors use Monte Carlo simulations to explore the stability of skyrmions against a ferromagnetic phase and their potential as single bits.

## Graphical abstract

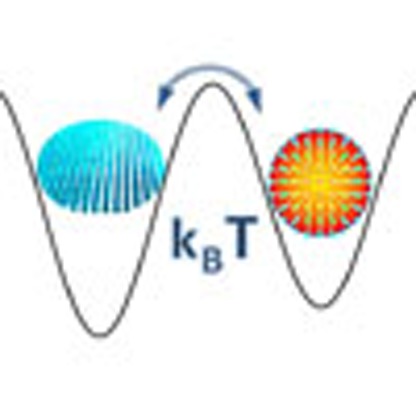

Magnetic skyrmions^1^ are vortex-like spin structures that have been observed at the nanometre scale in bulk non-centrosymmetric crystals^2^,^3^,^4^,^5^, in thin films of cubic helimagnets^6^,^7^,^8^ and in ultrathin films at surfaces^9^,^10^. Typically, skyrmions are formed due to the competition of the exchange and the Dzyaloshinskii–Moriya (DM) interactions^11^,^12^ in the presence of a magnetic field. Skyrmions exhibit particle-like properties and can emerge as isolated objects or in a close-packed lattice. The three-dimensional spins forming a skyrmion point in all directions wrapping a sphere, which gives rise to its topological stability against deformation into the ferromagnetic (FM) phase.

This robustness in combination with a small lateral size in the nanometre range has led to propositions to use skyrmions as bits of information in future data storage devices^13^,^14^. The realization of such a device requires the possibility to deliberately create and annihilate individual skyrmions as well as to manipulate their spatial position. There are several experimental and theoretical studies dealing with the motion of skyrmions driven by currents and temperature gradients^15^,^16^,^17^,^18^,^19^,^20^,^21^,^22^,^23^,^24^,^25^,^26^,^27^. In the continuum model, transitions between skyrmionic (Sk) and FM states are forbidden because of the topological protection. It is, however, commonly believed that in a real magnetic system with discrete atomic magnetic moments, the topological protection accounts for an energy barrier separating the two distinct states. This interpretation is justified by the fact that transitions between the Sk and FM states may be induced by a variation of the magnetic field and a coupling of the system to an external bath as has been shown both theoretically and experimentally in various studies^2^,^6^,^10^,^16^,^28^,^29^,^30^,^31^,^32^,^33^. In particular, the unwinding of skyrmion tubes into the helical spin state has been investigated with Monte Carlo (MC) simulations^30^ and the stochastic Landau-Lifshitz-Gilbert equation^33^ for chiral magnets. The main finding was that the annihilation process of these skyrmion tubes is governed by the dynamics of the formation of emergent magnetic monopoles and antimonopoles. Furthermore, the switching of skyrmions induced by local heating within a FM ultrathin magnetic film was studied with the stochastic Landau-Lifshitz-Gilbert equation as a function of heating time, intensity and area, recently^32^.

However, the shape of the energy landscape between the FM and Sk states and its impact on the transition dynamics are still unknown. The knowledge about the energy landscape of Sk systems is of huge importance for tailoring these nontrivial magnetic structures according to the requirements of data storage devices. While there are numerous MC studies^30^,^34^,^35^,^36^ dealing with the phase diagram, here we report on MC simulations regarding the kinetic properties of Sk and FM states in ultrathin magnetic films as a function of temperature and magnetic field. In our investigations, we find that the energy landscape of a Sk system is very different from that of magnetic atomic clusters and nanoislands exhibiting two-state behaviour with degenerate energy minima, which are symmetric with respect to the energy barrier^37^,^38^. The validity of the numerical results are tested by experimental data obtained on a Pd/Fe bilayer on an Ir(111) substrate. This allows us to propose a procedure for the determination of effective material parameters of real systems from quantification of MC mean lifetimes and magnetic fields using experimental data as shown exemplarily for a Pd/Fe bilayer on Ir(111).

## Results

### Monte Carlo analysis

For the sake of generality, we describe an ultrathin magnetic film by the standard effective Hamiltonian


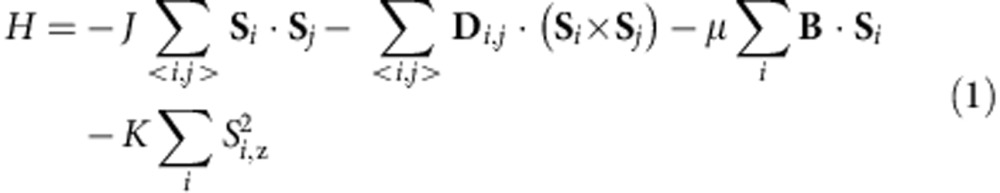


within the Heisenberg model. Therein, **S**_*i*_=**μ**_*i*_/*μ* is a three-dimensional magnetic moment of unit length, *K* describes the uniaxial perpendicular magnetic anisotropy and **B** is the uniform external magnetic field. *J* is the effective nearest-neighbour exchange integral and **D**_*i*,*j*_ is an effective DM coupling. The DM interaction arises, for example, due to the coupling of two atomic spins in a magnetic surface layer to a third atom of the supporting crystal, which exhibits a large spin–orbit coupling^39^. For symmetry reasons, we chose the DM vector **D**_*i*,*j*_ to be perpendicular to the vector connecting two spins **S**_*i*_ and **S**_*j*_ and to lie within the plane of the magnetic film^40^.

**Figure 1:**
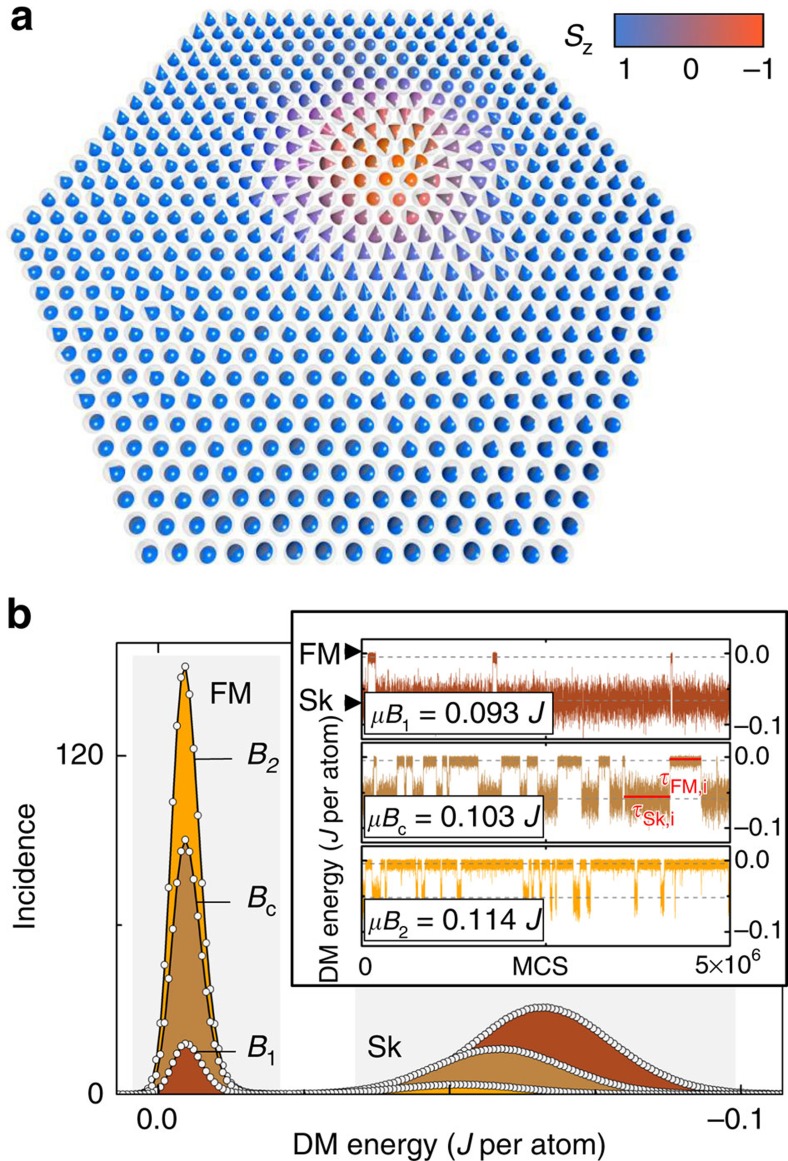
Thermally induced creation and annihilation of a single skyrmion. (**a**) Triangular spin lattice with a hexagonal boundary shape containing a single skyrmion at *k*_B_*T*=0.05 *J* and *μB*=0.1 *J*. (**b**) The inset displays the DM energy as a function of the Monte Carlo step (MCS) for different magnetic field strengths at *k*_B_*T*=0.61 *J* exhibiting a two-state behaviour due to the ongoing creation and annihilation of a single skyrmion. The size of the system is such that it contains a maximum of one skyrmion at a given MCS. The histogram of the DM energy shows two peaks corresponding to the FM and Sk states. The Sk and FM states are populated with equal probability at the critical field *B*_c_ and the areas underneath the peaks of the histogram are equal.

Systems consisting of up to 1,000 Heisenberg spins on a two-dimensional triangular lattice with a hexagonal boundary shape (Fig. 1a) using helical boundary conditions^41^ have been investigated by means of extended MC simulations (see the Methods section). Energy parameters typical for thin films showing Sk phases, that is, *D*/*J*≈0.3 and *K*≈0.1 *J* have been used^42^,^43^.

A thin magnetic film, which can be described by the equation (1), exhibits a rich phase space^6^. At low temperatures, the system exhibits a spin-spiral state in zero field and forms a temperature-dependent skyrmion lattice (Skx) or a skyrmion gas (Skg) in a perpendicular field. In a sufficiently large field, the system becomes fully polarized and transforms into a FM state. The simulations have been performed at fields and temperatures near the phase boundary separating the FM and Sk phases where individual skyrmions are formed. In this region of the phase space, the distance between skyrmions is much larger than the skyrmion diameter and the energies of the Sk and the FM states are nearly degenerate. Therefore, a thermally activated creation and annihilation of single skyrmions within the FM phase is expected. The size of the investigated system was adapted in such a way that only one single skyrmion was stochastically created and annihilated within the system near the critical field *B*_c_ separating the FM and Sk phases as a function of the time measured in MC steps (MCSs; see the Methods section). This switching behaviour was characterized by the calculation of the time dependence of the winding number oscillating between zero and unity and the DM energy exhibiting abrupt changes when a skyrmion is created or annihilated. As the DM energy is less dependent on local inhomogeneities of magnetic structures at high temperatures, we use it to plot and evaluate the telegraph noise due to the ongoing creation and annihilation of a skyrmion.

### Mean lifetimes

Figure 1b shows the histograms of the DM energies, which exhibit two peaks corresponding to the FM and Sk states. The peaks of the Sk states are much broader than those of the FM, which is due to thermally induced fluctuations in the skyrmion size. In addition, the peak of the Sk state shifts to higher energies with an increasing field indicating that the skyrmion equilibrium size decreases with the field^44^. As the probability to find the system in either state is equivalent to the area underneath the peaks, the areas are equal for both states at *B*_c_ only. The mean lifetimes 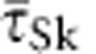 and 
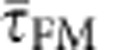
 cannot be deduced directly from the histograms but are rather determined as 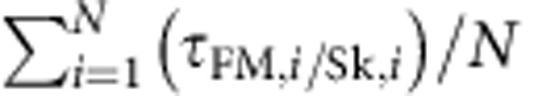 from the individual lifetimes *τ*_Sk,*i*_ and *τ*_FM,*i*_ shown in the inset of Fig. 1b. For this purpose, more than *N*=1,000 switching events have been recorded for each set of temperature and field to achieve reliable statistics. The inset in Fig. 1b shows the DM energy as a function of the MC time at three different magnetic fields for the temperature *k*_B_*T*=0.61 *J*. While the system is found with equal probabilities in the Sk and FM states at the critical field of *μB*_c_=0.103 *J*, the Sk state becomes preferred for fields *B*<*B*_c_ as shown for *μB*_1_=0.093 *J*. This population imbalance gets inverted in the range *B*>*B*_c_ at which the FM state becomes more stable as shown for *μB*_2_=0.114 *J*.

**Figure 2:**
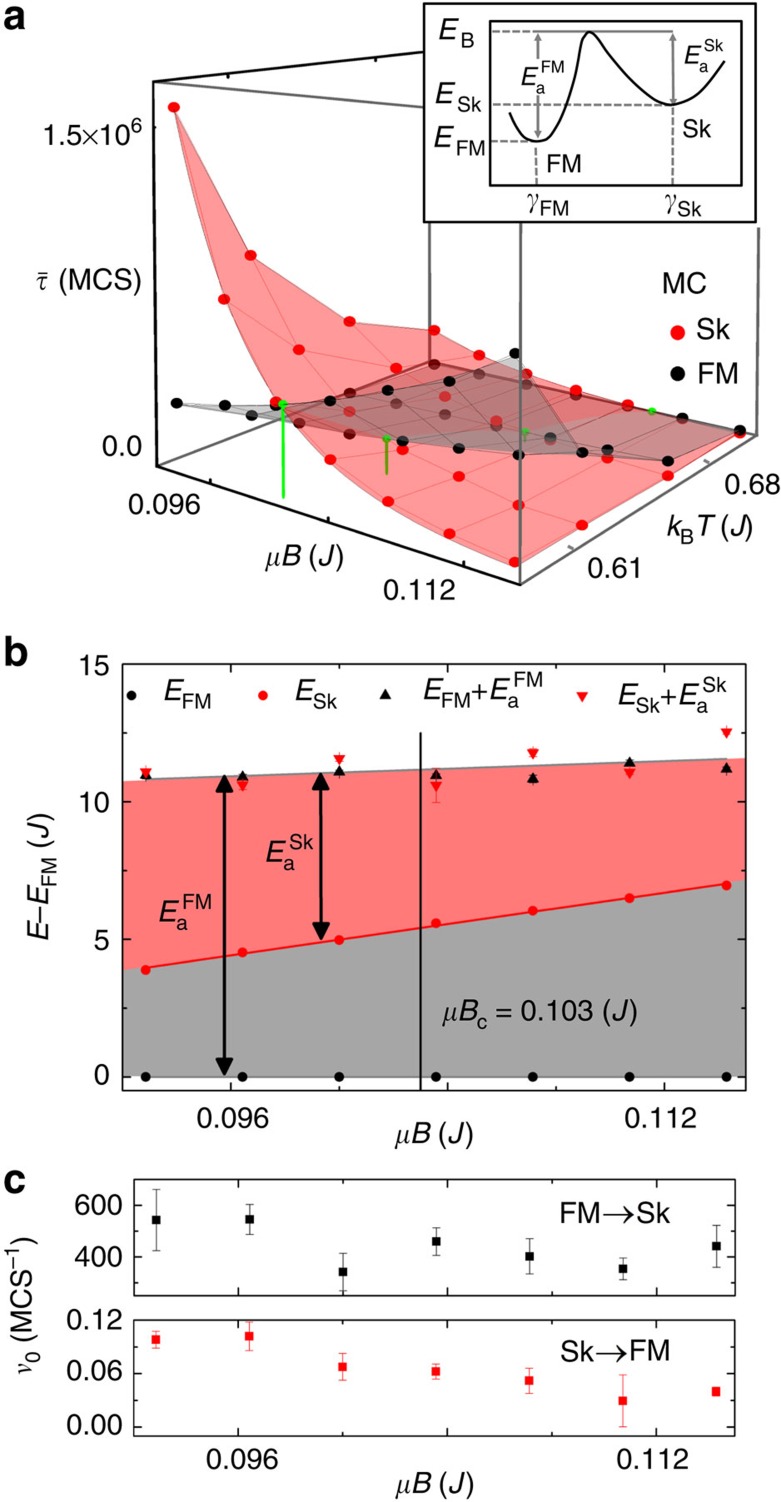
Stability properties of a single skyrmion. (**a**)Mean lifetimes of the Sk and FM states as a function of the magnetic field and the temperature. The red and black points are the results of MC calculations. The points of intersection 
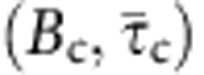
 are marked by green spheres. A cross-section of the data is shown in Fig. 3a with the same colour coding. The inset of **a** shows a sketch of the energy landscape. The energy minima *E*_Sk_ and *E*_FM_ of the Sk and FM states are separated by activation energies given by 
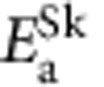
 and 
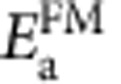
, respectively. (**b**) The energies *E*_Sk_ and *E*_FM_ of the Sk and FM states, respectively, as a function of the magnetic field for the temperature *k*_B_*T*=0.61 *J* alongside with the energy of the energy barrier *E*_B_, which is given by the sum of the energy levels and activation energies. (**c**) The attempt frequencies *ν*_0_.

Figure 2a shows the effect of both the magnetic field and the temperature on the mean lifetimes within the range *μB*=(0.093–0.114) *J* and *k*_B_*T*=(0.6–0.7)*J*. The red points give 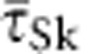 and the black points 
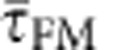
 as determined from the MC calculations. For a fixed temperature, the Sk state gets destabilized by an increasing field and, therefore, the mean lifetime of a skyrmion decreases. The FM state, in contrast, gets stabilized and the corresponding mean lifetime increases. Surprisingly, the mean lifetimes exhibit a very asymmetric behaviour with respect to the intersection point 
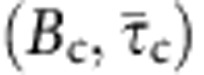
 showing that the Sk and FM states respond differently to changes in the magnetic field. Such an asymmetry can appear for several reasons including field dependent changes in the activation energy, in the energies of the Sk and FM states or in the dynamics.

### Energy landscape

To clarify the asymmetric behaviour of the mean lifetimes, particular attention has to be paid to the shape of the energy landscape and its dependence on the magnetic field in the vicinity of *B*_c_. As a starting point, we consider a simplified one-dimensional energy landscape (Fig. 2a, inset) with two energy minima *E*_Sk_ and *E*_FM_ of the Sk and FM states, which are separated by an energy barrier giving rise to the two activation energies 
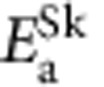
 and 
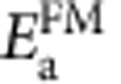
, respectively. The reaction coordinate *γ* describes the spin configurations between the two energy minima. The field dependences of *E*_FM_ and *E*_Sk_ can immediately be derived from the MC calculations. Figure 2b shows *E*_Sk_ with respect to *E*_FM_ at the temperature *k*_B_*T*=0.61 *J*. Both energies decrease linearly with the field, which is evident for the FM because of its direct Zeeman proportionality but is less trivial for the Sk state. The total energy of a single skyrmion may show some nonlinearity when examining large field ranges^45^, however, for a limited field range around *B*_c_, we find a linear dependence of its energy on the applied field. The FM state is energetically favoured over the Sk state over the full range of investigated fields. A linear extrapolation of *E*_FM_ and *E*_Sk_ to smaller fields yields a degeneracy at *μB*=0.065 *J*. Note that the skyrmion is more stable 
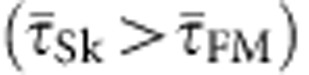
 for *μB*<0.103 *J*.

The energy of the separating barrier is more difficult to access because the intermediate spin configurations are yet unknown. However, 
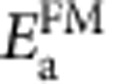
 and 
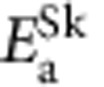
 can be derived from the temperature dependence of the mean lifetimes, which are found to decrease exponentially with the temperature allowing to describe them with the Arrhenius law by the functions


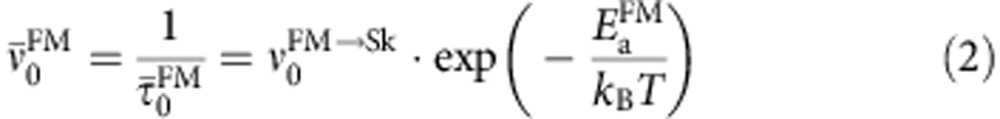



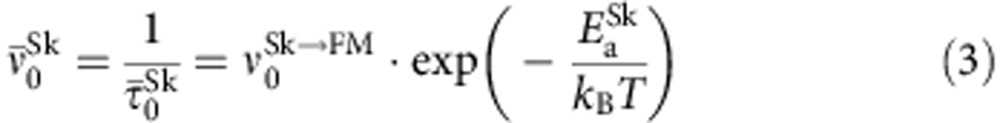


with the attempt frequencies 
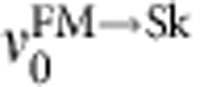
 and 
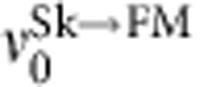
. The activation energies derived from fits of numerical data by equations (2) and (3) correspond to the vertical heights of the shaded areas in Fig. 2b. 
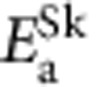
 and 
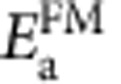
 both depend linearly on the magnetic field. 
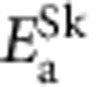
 decreases from 7.4 to 4.7 *J* in the investigated field range, while 
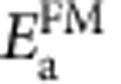
 increases only slightly and is about 11 *J*. The observation that 
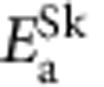
 responds more sensitive to changes in the field than 
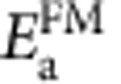
 may be explained by the fact that the magnetic microstructure of the FM state remains unchanged (up to fluctuations) with increasing field strength, while the size of an isolated skyrmion shows a field dependence^44^,^45^. A similar activated behaviour as described by equation (3) with a comparable value for 
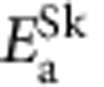
 was found for bulk Sk systems^33^.

The energy of the barrier state *E*_B_ is given by the sums of the energies of the FM and Sk states and the corresponding activation energies as 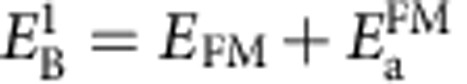 and 
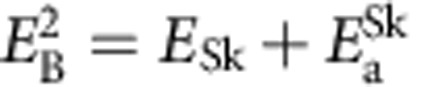
. The energies 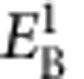 and 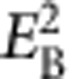, shown by the black and red triangles in Fig. 2b, coincide within the range of numerical accuracy indicating that the system passes the same barrier state for both transition directions justifying the one-dimensional picture of the energy landscape.

Summing up, we have found that for fields *B*<*B*_c_ the Sk state is more stable than its FM counterpart despite the fact that it is still energetically less favourable. Also, the activation energy of the Sk is smaller than that of the FM in this range. Thus, the intrinsic energy and the activation energy cannot be the dominating mechanism stabilizing the skyrmion. To shed light onto this finding, the attempt frequencies have been investigated. From the Arrhenius fit we find that the attempt frequencies are on the order of 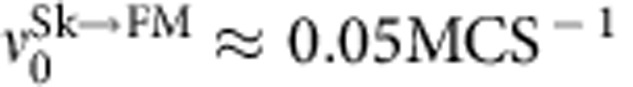 and 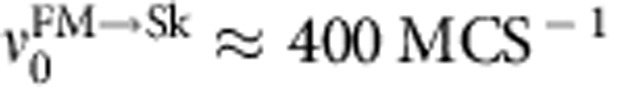 throughout the whole range of explored magnetic fields (Fig. 2c). We identify the large difference in the attempt frequencies as the reason for the stability of the Sk state for fields *B*<*B*_c_ in the present MC calculations.

To find a physical reason for the large difference of the attempt frequencies, the entropy of the system with and without a skyrmion^46^ has been estimated as presented in Supplementary Note 1 and Supplementary Fig. 1. The entropy difference is found to be of the order of 0.7–0.9 meV K^−1^. According to our analysis, the ratio of the attempt frequencies can be identified with this entropy difference^47^. Due to the higher entropy of the Sk state, the critical field *B*_c_(*T*) increases with the temperature (Supplementary Fig. 2). These data also imply that the energy minimum of the Sk state is broader than that of the FM state since the attempt frequencies describe in the first approximation the geometry of the energy landscape in the vicinity of the energy minima using Kramers' description of the escape rate in the regime of high damping^48^ (Supplementary Note 2).

### Deducing energy parameters

The calculations presented above concern a generic two-dimensional Sk system, which can effectively be described by an Hamiltonian of equation (1). In the following, we compare our numerical findings with experimental results obtained for Pd/Fe/Ir(111)^10^. The experimental investigations on Pd/Fe/Ir(111) were performed at a temperature of 4.2 K with a spin-polarized scanning tunnelling microscope (SP-STM) using a Cr bulk tip. The system exhibits a spin-spiral state at zero magnetic field at low temperatures. In a perpendicular magnetic field, the system adapts a two-level behaviour: only Sk and FM configurations remain stable^10^. The temperature is such that the thermal energy alone is insufficient to go from the FM to the Sk state at moderate field values between 2.5 T and 4.5 T. Instead, the additional energy from the spin-polarized tunnel current is needed to overcome the energy barrier and to reach the other energy minimum. Single skyrmions that are pinned to atomic defects in the FM phase were observed at fields of 3.5–4 T. Within this field range, using a spin-polarized tunnel current *I*=100 nA and an applied voltage of *U*=±600 mV, a stochastic switching between the Sk and the FM states was induced and the corresponding telegraph signal in the differential conductance has been recorded^10^.

To justify a comparison between these experimental data and the present MC calculations, particular attention has to be paid to the origin of the excitations leading to the spontaneous switching between the two states. In standard MC simulations, the system under investigation is in equilibrium with a thermal bath, which is described by the energy *E*_b_ being an essential part of the Boltzmann probability 
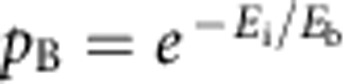
. The exact origin of *E*_b_ is never addressed and is traditionally attributed to the thermal energy *E*_b_=*k*_B_*T*. In the present experimental system, the spontaneous switching between the two states is not purely induced by thermal energy. Instead, the dominant mechanism was found to be the injection of additional energy due to tunnelling electrons from the tip of a spin-polarized scanning tunnelling microscopy^10^. Here we assume that the MC temperature includes both thermal and electronic excitations. Note also that the experiments were performed on skyrmions located at local atomic defects, which were neglected within the MC simulations.

**Figure 3:**
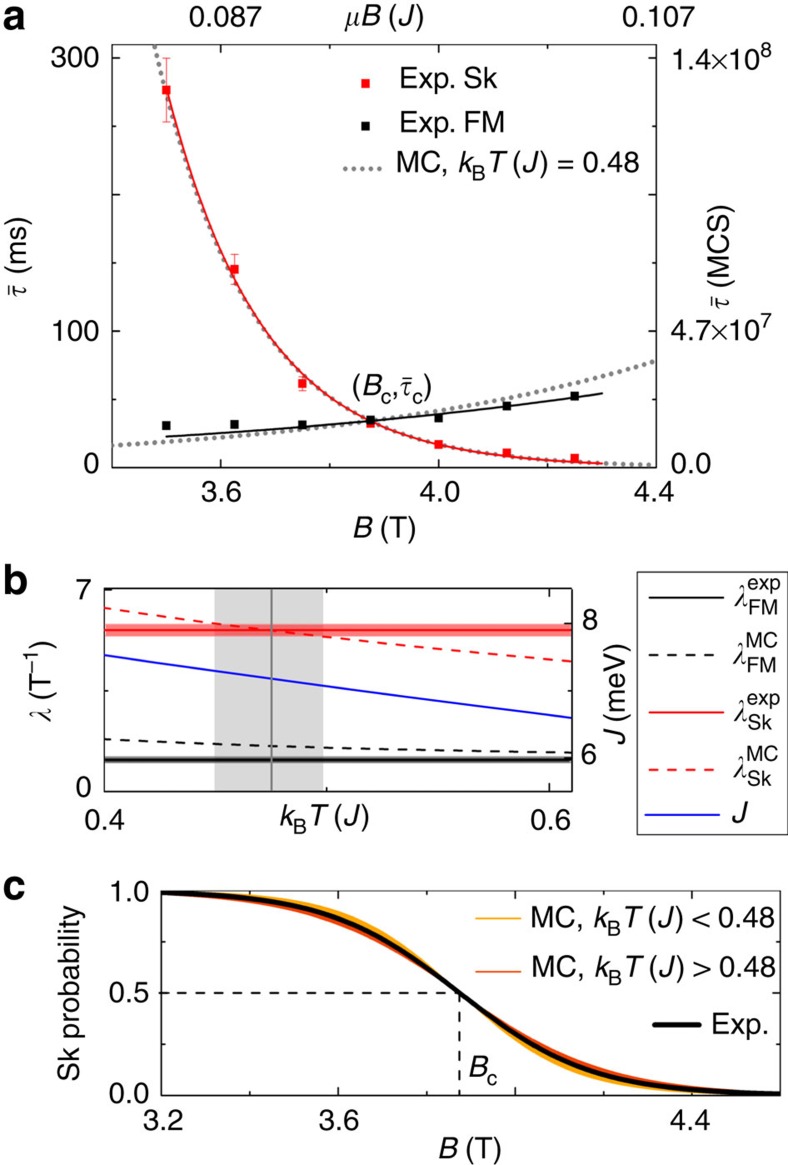
Calibration of MC results with experimental data obtained on Pd/Fe/Ir(111). (**a**) The mean lifetimes obtained from experiments on Pd/Fe/Ir(111) as a function of the magnetic field *B* in combination with mean lifetimes obtained from MC calculations. The MC data are linearly rescaled to the experimental data such that the critical points given by 
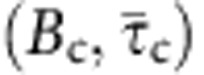
 coincide. The lifetimes are described by exponential functions 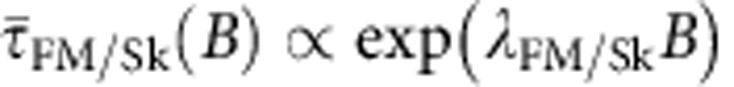. (**b**) *λ*_FM/Sk_ for the experimental data (exp) and MC data (MC). The values 
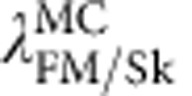
 depend on the MC temperature used. The best correspondence between experiment and MC is found using the MC temperature *k*_B_*T*≈0.48 *J* marked by the vertical grey line. The calibration of the MC mean lifetimes at this temperature with the experimental lifetimes provide an absolute value for the exchange parameter of *J*=7.1±0.2 meV. (**c**) The skyrmion probability 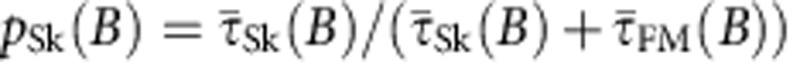.

Figure 3a shows experimentally obtained mean lifetimes of the Sk and FM states as a function of *B* for the bias voltage of *U*=−600 mV. The functions show a very good qualitative agreement with the theoretically obtained data of Fig. 2a. In agreement with the MC data, the lifetimes are strongly asymmetric with respect to the critical field *B*_c_≈3.9 T at which the lifetimes are equal. This finding allows us to compare the experimental and MC mean lifetimes. More than that, we propose to calibrate our MC results using the experimental data by linearly rescaling 
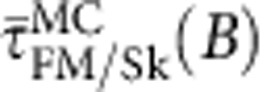
 in such a way that the MC intersection point 
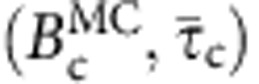
 coincides with that found experimentally. Now, the task remains to determine the correct MC temperature at which the field-dependent mean lifetimes best fit to the experimental data. To find the required MC temperature, 
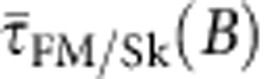
 was described by exponential functions of the form


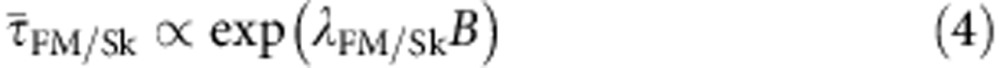


with the fit parameters *λ*_FM/Sk_ as proposed in ref. 33 for the Sk state. The horizontal black and red lines in Fig. 3b show the values 
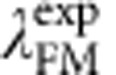
 and 
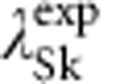
 obtained from fits to the experimental data. The thickness of these lines gives the uncertainty. For the MC data, the values 
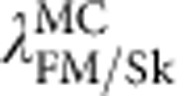
 depend on the MC temperature and are shown by the red and black dashed lines in Fig. 3b. The curves 
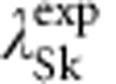
 and 
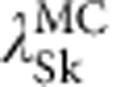
 intersect at *k*_B_*T*=0.48 *J*, while no intersection can be observed for 
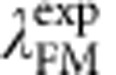
 and 
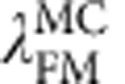
 within the explored MC temperature range. We conclude that the best agreement between experiment and MC simulations is obtained for the MC temperature *k*_B_*T*≈0.48 *J*. Note that for *k*_B_*T*<0.58 *J*, the MC mean lifetimes were extrapolated from the temperature-dependent calculations because of insufficient MC statistics in direct simulations. The feasibility of the proposed calibration method has been confirmed by the calculation of the Sk probability 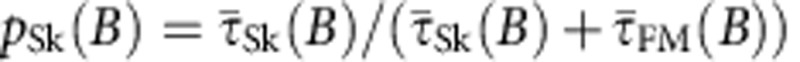 (Fig. 3c), which is 0.5 at the critical field *B*_c_ and approaches 1 for small and 0 for large fields. The experimentally and numerically determined curves show a good agreement.

From the correspondence between the experimental and the MC time at *B*_c_, the physical timescale is immediately set such that 1 MCS corresponds to 2 × 10^−9^ s. From the knowledge of the experimental *B*_c_, the MC field is derived by the scaling factor 
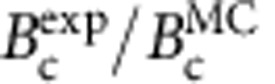
. Taking the magnetic moment *μ*=3 *μ*_B_ for Pd/Fe/Ir(111)^42^ into account, all coupling constants can naturally be determined. According to the described calculations the nearest-neighbour effective exchange interaction for Pd/Fe/Ir(111) equals *J*=7±0.2 meV per bond, while the DM parameter is *D*=2.2 meV per bond. Our value for *D* is in a good agreement with those given in refs 42, 43, 49, while our value for the effective nearest-neighbour interaction is somewhat smaller compared with the values for the nearest-neighbour interaction in refs 42, 43. This difference in *J* may be explained by the fact that compensating antiferromagnetic interactions between more distant neighbours^42^,^43^ are mapped onto our effective nearest-neighbour exchange parameter. From the above considerations, the activation energies between single skyrmions and the FM phase in Pd/Fe/Ir(111) are on the order of 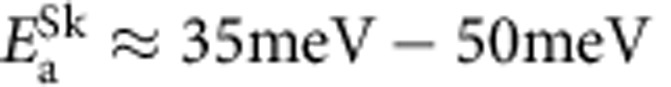 and 
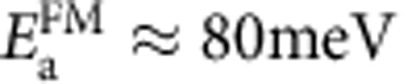
 at the temperature *k*_B_*T*=0.48 *J*. This is somewhat smaller than activation barriers observed for nanoscale Fe/W islands of comparable size^50^ (≈100 atoms) to the skyrmions.

## Discussion

The overall good agreement suggests that the present MC simulations are suited to describe Sk systems and capture the physics of the creation and annihilation of individual skyrmions. The results of our general MC treatment reveal several important peculiarities of the switching between single Sk and FM states. The most important finding is that in contrast to other nanomagnets the field-dependent mean lifetimes of the Sk and FM states are strongly asymmetric. Our analysis reveals that the stability of skyrmions is hidden in the dynamics and the geometry of the energy landscape. Particularly, the attempt frequency of the skyrmion annihilation is orders of magnitudes smaller than that of the skyrmion creation due to a more shallow shape of the potential. The good agreement of these general conclusions with the experimental data on the Pd/Fe bilayer on Ir(111) allow us to make predictions about the applicability of field stabilized skyrmions in thin magnetic films for data storage devices. First, to be able to populate the two states corresponding to 0 and 1 with an equal probability the applied magnetic field has to be close to *B*_c_. Second, to ensure the stability of the two states of a bit, the energy barrier and hence the activation energy has to be optimized. For the particular example of Pd/Fe Ir(111), we find that the upper limit for 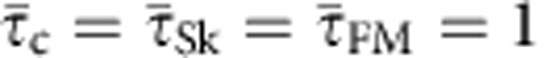 year at *B*_c_ corresponds to a temperature of *T*=19 K (Supplementary Note 3; Supplementary Fig. 3). Thus, a device on the basis of this material will be able to operate at the temperature of liquid He only. To increase the operation temperature, the activation energies need to be larger while leaving the *D*/*J* ratio constant. We speculate that this can be achieved by a coherent increase of both energy parameters by, for example, using multilayers to increase the number of nearest-neighbours and at the same time the number of interfaces, which are necessary for a strong DM coupling. With a *J*_eff_=100 meV per bond one gets stable skyrmions at 300 K. We anticipate that our studies will help to systematically tailor magnetic Sk systems for future information storage applications.

## Methods

### Simulation details

All Monte Carlo calculations presented in the main text were performed with the Metropolis algorithm on a two-dimensional triangular lattice of *N*=631 sites with a hexagonal boundary shape. Each MC step consisted of *N* trial manipulations. The choice of the system size was made such to best meet the requirements of the problem at hand and is discussed within the section Choice of system size below. Each site was populated by a classical Heisenberg moment 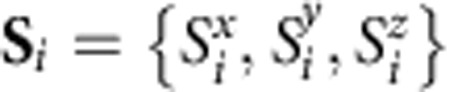 of unit length **S**_*i*_=**μ**_*i*_/*μ*. Helical boundary conditions were used in order to minimize finite size effects.

We considered a Hamilton operator of the form


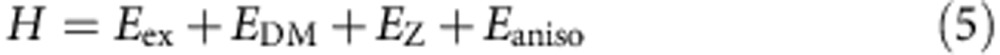


including the exchange interaction, DM interaction, Zeeman energy and anisotropy energy. The exact terms of these energies are given in the main text. For all simulations presented in the main text, system parameters *D* and *J* with a ratio of *D*/*J*=0.32 and an easy perpendicular anisotropy axis of *K*=0.07 *J* were used. We confirmed that this system shows a phase space behaviour qualitatively similar to the observations made in ref. 6.

**Figure 4:**
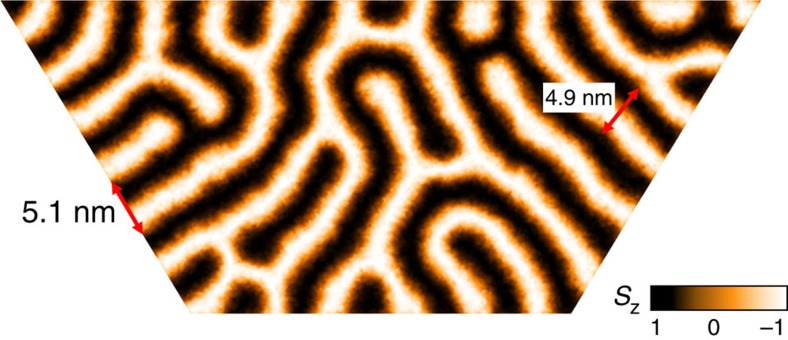
Spin-spiral period. Density plot of spin-spirals obtained with MC simulations in zero field at a temperature of *k*_B_*T*=0.06 *J*. The spin-spiral period is determined to be *λ*≈5 nm considering the lattice constant *a*=2.7 Å of Fe/Ir(111)^9^.

In zero field at *k*_B_*T*=0.06 *J*, the system exhibits spin-spirals with a period of *λ*≈5 nm (Fig. 4) assuming the lattice constant *a*=2.7 Å of Fe/Ir(111)^9^. This value for the spin-spiral period is somewhat smaller than the ones found experimentally for Pd/Fe/Ir(111), which are on the order of 6–7 nm (ref. 10). To determine the spin-spiral period, we chose a system of ∼3 × 10^4^ Heisenberg spins on a triangular lattice with open boundary conditions to achieve a sufficient number of spin spirals. The temperature was successively decreased from *k*_B_*T*=0.61 *J* for an arbitrary initial spin configuration to *k*_B_*T*=0.06 *J* using 40 temperature steps with 10^5^ MCS each. Note that the boundary shape of the lattice used for these MC simulations was adapted to resemble the typical island shapes found in the experimental investigations of Pd/Fe/Ir(111)^10^.

**Figure 5:**
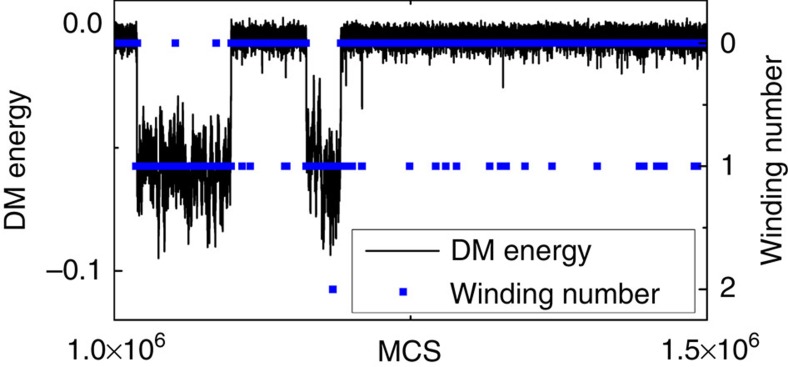
DM energy and winding number. The DM energy and the corresponding winding number as a function of the time given in MC steps for *k*_B_*T*=0.61 *J* and *μB*=0.1 *J* and system size *N*=631 exhibiting a two-state behaviour due to the ongoing creation and annihilation of a single skyrmion.

The focus of the investigations in the main text was laid upon the field region in which the Sk and FM states become energetically nearly degenerate and thus allow for a thermally activated spontaneous creation and annihilation of individual skyrmions in the FM phase as a function of the time given in MCS. This switching behaviour was characterized by the winding number and the DM energy shown exemplarily in Fig. 5 for *k*_B_*T*=0.61 *J* and *μB*=0.1 *J* and the system size *N*=631. The DM energy exhibits a two-level behaviour while the winding number adopts discrete natural number values between zero and two for these parameters within this range of MCS. The winding number switches predominantly between zero and one corresponding to the two levels in the DM energy. However, local fluctuations add or remove a skyrmion to the winding number which we neglect when counting the total number of skyrmions within the system. Hence, we use for convenience the DM energy to investigate the switching behaviour and to determine the mean lifetimes since the DM energy is less sensitive to local fluctuations.

**Figure 6:**
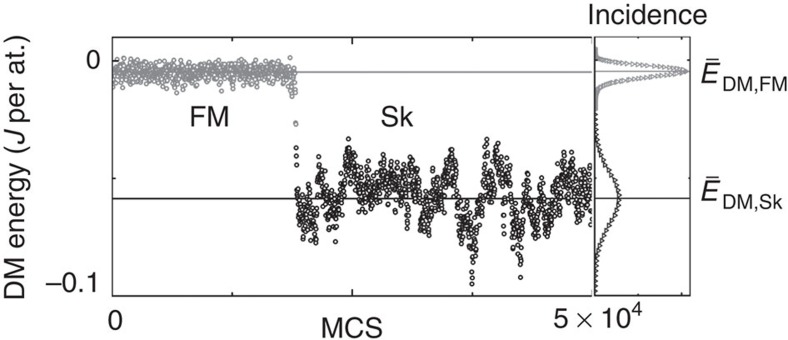
Identification of a switching event. DM energy as a function of the time given in MC steps for *k*_B_*T*=0.61 *J*, *μB*=0.1 *J* and a system size *N*=631 exhibiting a two-state behaviour due to the ongoing creation and annihilation of a single skyrmion. The frequency distribution of the DM energies exhibits two peaks whose positions 
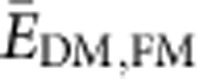
 and 
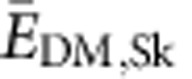
 are marked by the grey and black horizontal lines. A switching event is identified by the coincidence when the DM energy subsequently crosses these two energies from either direction.

In the following, we comment on the method by which the switching events between the Sk and FM states were identified. The frequency distribution of the DM energy at a particular temperature and magnetic field exhibits two peaks corresponding to the FM and Sk states as discussed in the main text and shown in Fig. 6 for *k*_B_*T*=0.61 *J*, *μB*=0.1 *J* and a system size *N*=631. The peak positions are located at the mean DM energies 
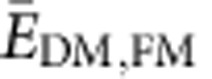
 and 
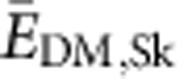
 of the FM and Sk states as shown by the horizontal grey and black lines. The switching events are identified by finding the coincidences at which the DM energy subsequently crosses the energies 
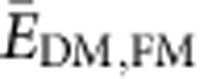
 and 
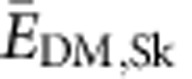
 in either direction. In Fig. 6, a switching event from the FM to the Sk state is shown.

### Choice of system size

**Figure 7:**
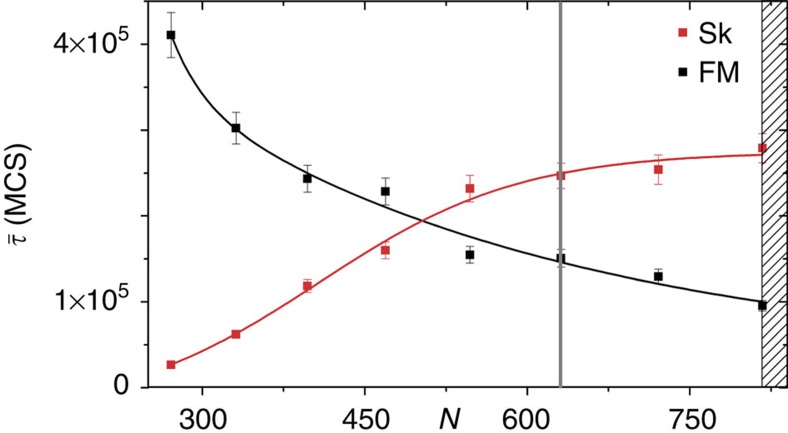
Mean lifetimes as a function of the system size. The mean lifetimes 
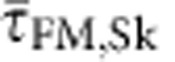
 as a function of the system size *N* for *k*_B_*T*=0.61 *J* and *μB*=0.1 *J*. Since triangular lattices with a hexagonal boundary shape are considered, discrete values of *N* are possible only. The red and black points mark the mean lifetimes for the values of *N* that comply with this constriction. The shaded area to the right side marks the region in which the system size is large enough to allow two skyrmions to be present simultaneously. We chose the system size *N*=631, which is marked by the grey vertical line for the simulations presented in the main text.

The total number of skyrmions that can be present in the system simultaneously depends on the system size *N*. Therefore, the system size has an influence on the stability of an individual skyrmion. Figure 7 shows the mean lifetimes of the FM state and an individual skyrmion as a function of the system size for *k*_B_*T*=0.61 *J* and *μB*=0.1 *J*. We restricted the system to geometries with a hexagonal boundary shape leaving only discrete possible values for *N*. The red and black points mark the mean lifetimes for the values of *N* that comply with this constriction. The system can contain a maximum of one skyrmion for relatively small system sizes *N*<800. In the range 500<*N*<800, the single skyrmion is the more probable state compared to the ferromagnet. For system sizes *N*<500, this stability relation is inverted and the ferromagnet is the more probable state. This behaviour may be ascribed to two effects. First, the skyrmion is restricted to a smaller region with a reduction of the system size providing less space to evade local perturbations. Second, the system eventually becomes too small to allow the formation of a whole skyrmion. In systems larger than *N*≈800, multiple skyrmions may be present within the system at the same time. Since we wanted to investigate the stability properties of an individual skyrmion, we chose a system size of *N*=631 marked by the grey vertical line, which is close to the region allowing for multiple skyrmions.

## Additional information

**How to cite this article:** Hagemeister, J. *et al*. Stability of single skyrmionic bits. *Nat. Commun.* 6:8455 doi: 10.1038/ncomms9455 (2015).

## Supplementary Material

Supplementary Information Supplementary Figures 1-3, Supplementary Notes 1-3 and Supplementary References
