# Supplementary material for: Stability of single skyrmionic bits
Source: Nat Commun. 2015 Oct 14;6:8455. doi: 10.1038/ncomms9455 (PMC4634135; doi:10.1038/ncomms9455)
Supplement: Supplementary Information — Supplementary Figures 1-3, Supplementary Notes 1-3 and Supplementary References [file ncomms9455-s1.pdf]

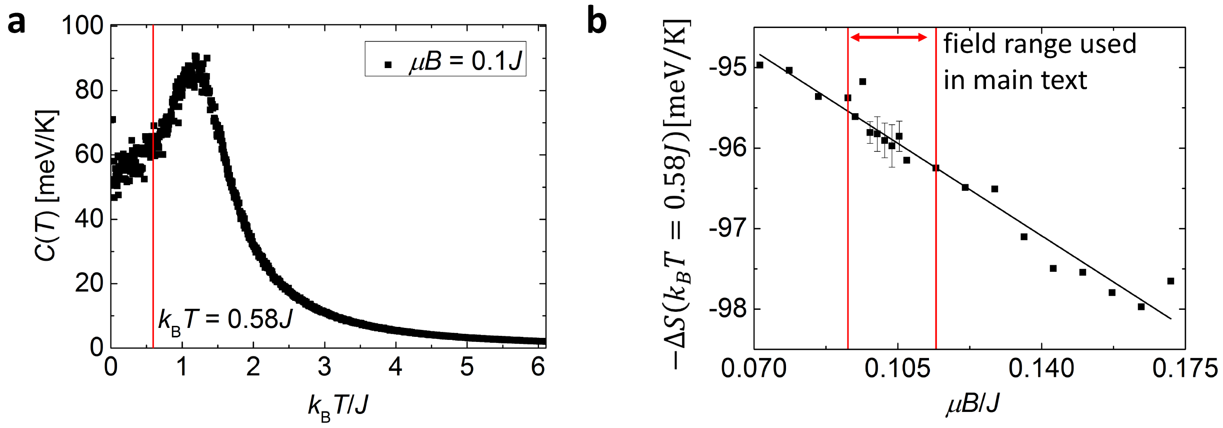

Supplementary Figure 1 | **Heat capacity and entropy.** (**a**) Heat capacity $C(T)$ as a function of the temperature for the magnetic field $\mu B = 0.1J$. (**b**) Entropy difference $-\Delta S$ as a function of the magnetic field.

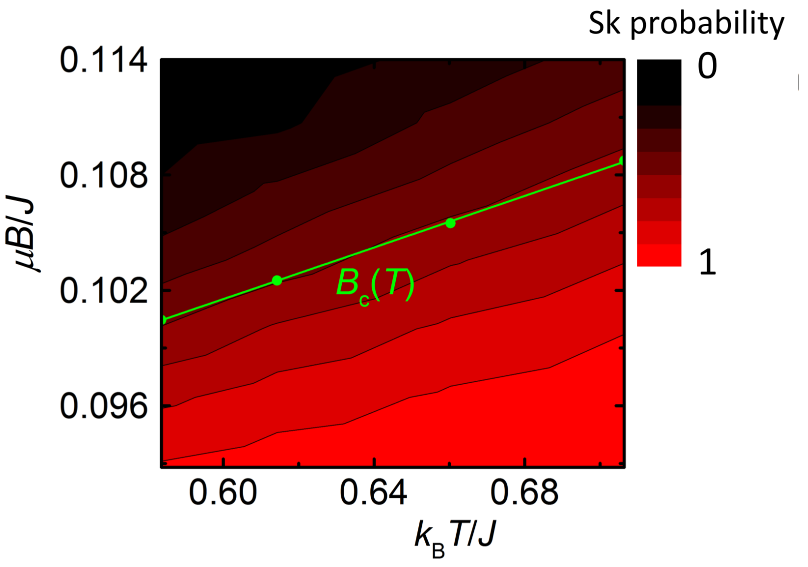

Supplementary Figure 2 | **Skyrmion probability and critical magnetic field**. The critical magnetic field derived from MC simulations as a function of the temperature. The skyrmion probability $\overline{\tau}_{\rm Sk}/(\overline{\tau}_{\rm Sk} + \overline{\tau}_{\rm FM})$ is color coded in red to black.

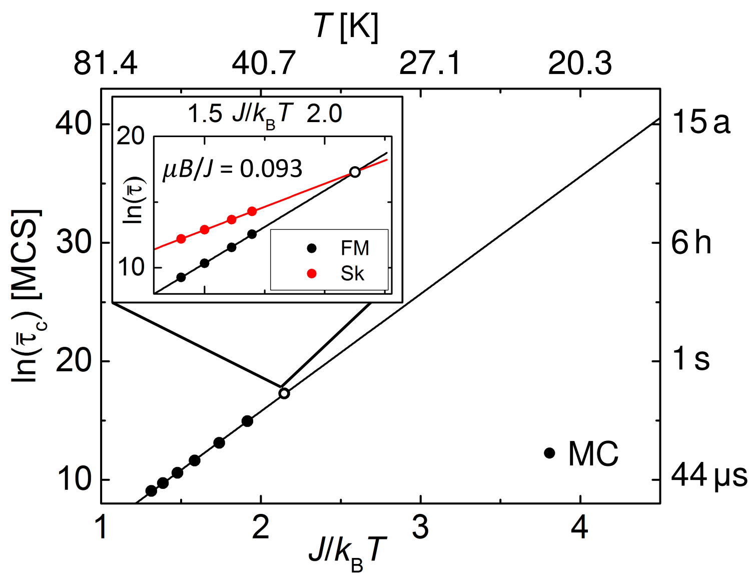

Supplementary Figure 3 | **Temperature dependence of $\overline{\tau}_{\mathrm{c}}$.** The mean lifetime $\overline{\tau}_{\mathrm{c}} = \overline{\tau}_{\mathrm{FM}}(B_{\mathrm{c}}) = \overline{\tau}_{\mathrm{Sk}}(B_{\mathrm{c}})$ as a function of the inverse temperature $J/k_{\mathrm{B}}T$ obtained from MC simulations. A temperature lower than $19\,\mathrm{K}$ is needed to achieve mean lifetimes on the order of years in the system Pd/Fe/Ir(111).

Supplementary Note 1 | Entropy

In the following, we comment on the entropy of the system which is being discussed in the main text and draw a connection to the temperature dependence of the critical field B_c.

Ezawa[1] estimated the entropy of an extended thin magnetic film exhibiting skyrmions and used it to derive a phase diagram spanned by the temperature and the magnetic field. Particularly, the critical magnetic field separating the skyrmionic and ferromagnetic phases was found to increase with the temperature due to a higher entropy of the skyrmion phase. We observed a similar behavior for the critical field B_c as shown in Supplementary Figure 2.

Ezawa[1] treats the entropy of the extended system adapting at maximum N skyrmions by using the formula for n subset-element combinations $N!/n!(N-n)!$. Hence, skyrmions here are treated as an ensemble of quasiparticles. In our investigation, a single skyrmion is an extended object with internal degrees of freedom. Therefore, another approach for the estimation of the entropy for the system discussed in the main text is needed.

We determine the entropy from thermodynamic principles from the heat capacity C as Ramirez *et al.*[2]. The heat capacity is calculated in the Monte-Carlo simulation by

$$C = \frac{< E^2 > - < E >^2}{k_\mathrm{B} T^2}$$

as a function of the temperature for various magnetic fields. For a given magnetic field, the tem-

perature was reduced from $k_BT = 6\,J$ (paramagnetic disordered state) to $k_BT = 0.01\,J$ (ordered equilibrium state) in 1000 steps and $2 \cdot 10^4$ Monte-Carlo steps were done at each temperature step. Supplementary Figure 1a shows the heat capacity as a function of the temperature exemplarily for the magnetic field $\mu B = 0.1\,J$.

The entropy $S(T)$ can then be determined by evaluating the following integral:

$$S(T) = \int_{T'=0}^{T'=T} \frac{C(T')}{T'} dT'.$$

In Monte-Carlo simulations we face the problem that the temperature is always a value greater than zero and therefore, for $k_\mathrm{B}T < 0.58\,J$ the integral cannot be properly evaluated. However, we have access to the entropy difference between a low temperature T_0 and a high temperature T_∞ at which the sample is disordered.

$$\Delta S = \int_{T'=T}^{T'=T_\infty} \frac{C(T')}{T'} dT'$$

The total magnetic entropy of N independent quantum spins is known to be $S = R \cdot \ln(2J+1)$ with J the total angular momentum. For classical spin systems this value corresponds to $S = R \cdot \ln 4\pi$ as stated by McMichael *et al.*[3]. Hence, the magnetic entropy per spin can reach a maximum of $S/R = \ln 4\pi \approx 2.48$. In the limit of strong interactions this value might be strongly reduced. However, we can take the limit of $\ln 4\pi$ as a total spin entropy at an infinite temperature. Hence, while we cannot

give the absolute values of entropy at finite temperatures, we can calculate $\Delta S = S(T_\infty) - S(T)$ as a function of external magnetic field. Supplementary Figure 1b shows $-\Delta S(k_\mathrm{B}T = 0.58\,J, B)$ in the broad range of magnetic fields for $k_\mathrm{B}T = 0.58\,J$, which lies below the ordering temperature. As one can see from this figure, $\Delta S(B)$ linearly increases with increasing field ($-\Delta S(B)$ linearly decreases). This means that the entropy of the ordered state $S(T, B)$ decreases with increasing field as the total entropy is $S(T, B) = S(T_\infty) - \Delta S$. In other words, the skyrmionic state ($B < B_\mathrm{c}$) possesses a larger entropy than the ferromagnetic state.

Since the influence of the entropy becomes more prominent for higher temperatures, the stability of a skyrmion increases at a given magnetic field with the temperature and hence, $B_\mathrm{c}(T)$ should also increase with the temperature. We estimate the entropic contribution due to the formation of a single skyrmion to be on the order of $0.7\,\mathrm{meV/K}$ which is the entropy difference between the magnetic fields $B = 1.1B_\mathrm{c}$ and $B = 0.9B_\mathrm{c}$.

This result about the entropy difference is in good agreement with considerations using the Eyring equation [4,5]

$$\nu = \nu_0 \exp(\frac{\Delta S}{k_\mathrm{B}}) \exp(-\frac{\Delta U}{k_\mathrm{B}T}) \qquad (1)$$

describing the transition rate ν by a prefactor ν_0 and the entropy and internal energy differences ΔS and ΔU going from the initial to the transition state. Applying the Eyring equation to the skyrmionic system, one obtains the attempt frequencies ν_Sk and ν_Sk of the Sk and FM states as

$$\nu_{\mathrm{Sk}} = \nu_0 \exp(\Delta S_{\mathrm{Sk}}/k_{\mathrm{B}}) \exp(-\frac{\Delta U_{\mathrm{Sk}}}{k_{\mathrm{B}}T}) \tag{2}$$

$$\nu_{\mathrm{FM}} = \nu_0 \exp(\Delta S_{\mathrm{FM}}/k_{\mathrm{B}}) \exp(-\frac{\Delta U_{\mathrm{FM}}}{k_{\mathrm{B}}T}) \tag{3}$$

At B_{c}, the transition rates are equal ($\nu_{\mathrm{Sk}} = \nu_{\mathrm{FM}}$) and therefore

$$\Delta U_{\mathrm{Sk}} - \Delta U_{\mathrm{FM}} = T(\Delta S_{\mathrm{Sk}} - \Delta S_{\mathrm{FM}}) \tag{4}$$

which is equivalent to the statement that the Gibbs energies of the FM and Sk states are identical. The differences $\Delta U_{\mathrm{Sk}} - \Delta U_{\mathrm{FM}}$ and $\Delta S_{\mathrm{Sk}} - \Delta S_{\mathrm{FM}}$ are equal to the differences of the internal energies and entropies of the two states. The energy difference has been found to be $\approx 6\,J$ at B_{c} from Figure 2b of the main text. This leads to an entropy difference of $\approx 0.9\,\mathrm{meV/K}$ between the FM and Sk states with ($S_{\mathrm{Sk}} > S_{\mathrm{FM}}$) at the temperature $k_{\mathrm{B}}T/J = 0.58$. This temperature was also investigated in the main text. The entropy difference is close to the one stated above of $0.7\,\mathrm{meV/K}$.

Moreover, we can show a direct link between the dependence of the critical magnetic field as a function of the temperature $(B_{\mathrm{c}}(T))$ and the entropy difference between the Sk and FM states. From equation 4 follows

$$E_{\mathrm{FM}}(B_{\mathrm{c}}, T) - E_{\mathrm{Sk}}(B_{\mathrm{c}}, T) = T(S_{\mathrm{FM}}(B_{\mathrm{c}}, T) - S_{\mathrm{Sk}}(B_{\mathrm{c}}, T)) \tag{5}$$

with E_{FM}, E_{Sk} and S_{FM}, S_{Sk} the energies and entropies of the FM and Sk states. The difference of the activation energies is equal to the difference of the energies of the FM and Sk states because the transition state is the same for both directions as shown in the main manuscript for the present system.

Differentiating with respect to the temperature while assuming that the entropy difference is in a first approximation independent of the temperature $(\Delta S(B_{\mathrm{c}}, T) = S_{\mathrm{FM}}(B_{\mathrm{c}}, T) - S_{\mathrm{Sk}}(B_{\mathrm{c}}, T)) = \Delta S(B_{\mathrm{c}})$ yields

$$\frac{\partial}{\partial T}(E_{\mathrm{FM}} - E_{\mathrm{Sk}}) = \Delta S(B_{\mathrm{c}}) \tag{6}$$

It is reasonable to assume that the equilibrium size of a skyrmion at the critical field is approximately independent of the temperature. Therefore, the change of difference of the energy levels with the temperature is dominated by the Zeeman energy and hence

$$\frac{\partial}{\partial T}(E_{\mathrm{FM}} - E_{\mathrm{Sk}}) \approx \frac{\partial}{\partial T}\Delta E_{\mathrm{Z}}(B_c) \approx \Delta M_{\mathrm{z}} \frac{\partial}{\partial T} B_{\mathrm{c}} \tag{7}$$

The difference of the z-component of the magnetisation between the two states can be derived from the MC simulations as $\overline{\Delta M_{\mathrm{z}}} \approx 120\,\mu$. The slope of the change of the critical field with respect to the temperature can be taken from Supplementary Figure 2 and one obtains

$$|\overline{\Delta M}_{\mathrm{z}} \frac{\partial}{\partial T} B_{\mathrm{c}}| \approx 0.7\,\mathrm{meV/K} \tag{8}$$

Thus, different ways lead to similar values within the range of $(0.7 - 0.9)\,\mathrm{meV/K}$ as the entropy difference between the Sk and FM states. Hence, we conclude that the skyrmion stability is mainly defined via the entropic contribution.

Supplementary Note 2 | Damping regime

The interpretation concerning the shape of the potential wells of the skyrmionic and ferromagnetic states are motivated by the formula for the escape rate r for a system with a viscosity η and an energy barrier Δ in the regime of a high damping[6]

$$r = \frac{\omega\omega'}{\eta} \exp(-\Delta/k_{\mathrm{B}}T) \tag{9}$$

Therein, ω and ω' approximate by harmonic potentials the energy landscape around the energy minimum and the barrier respectively. So, if the barrier height and shape ω' is identical on the ways back and forth which is a reasonable assumption because the identity of the barrier height has been shown, the difference in total frequency $\omega \cdot \omega'/\eta$ can only be explained by the shape of the energy minima. To be able to apply the Kramers' theorem one has to satisfy two conditions (i) the energy barrier has to be significantly higher than the thermal energy and (ii) the system has to

be in the regime of intermediate to high damping. The energy barriers for the skyrmionic system presented in the manuscript have a height of about $(5-10)\,J$ which is by about a factor of ten larger than the thermal energy of $k_B T \approx 0.6\,J$. Hence, the condition (i) is satisfied. The determination of the correct damping regime (ii) is more subtle. There are strong arguments indicating that we are working in the regime of high damping. It has been shown for an ensemble of single domain particles that the results of the Landau-Lifshitz-Gilbert equation and a classical heat-bath Monte-Carlo scheme coincide in the regime of high damping[7]. We believe that this provides a good approximation to our MC simulations even though the skyrmionic system is more complex. In other words, the regime of high damping is imposed by the simulation method.

Supplementary Note 3 | Predictions about stability bounds of skyrmions in Pd/Fe/Ir(111)

In the main text, we present a prediction for individual skyrmions in Pd/Fe/Ir(111) which states that mean lifetimes larger than $\overline{\tau}_{\mathrm{Sk}} = \overline{\tau}_{\mathrm{FM}} = 1\,\mathrm{year}$ are obtained for temperatures below $19\,\mathrm{K}$. In the following, we comment on the method that was applied to obtain this prediction.

Using the results of the MC simulations, we investigated the dependence of the mean lifetime $\overline{\tau}_{\mathrm{c}} = \overline{\tau}_{\mathrm{FM}}(B_{\mathrm{c}}) = \overline{\tau}_{\mathrm{Sk}}(B_{\mathrm{c}})$ at the critical field B_{c} as a function of the inverse temperature $J/k_{\mathrm{B}}T$ finding an exponential dependence as shown in Supplementary Figure 3. The data points were obtained from the field and temperature dependent mean lifetimes presented in the main text in Figure 2a. Considering a constant magnetic field, the mean lifetimes $\overline{\tau}_{\mathrm{Sk}}(T)$ and $\overline{\tau}_{\mathrm{FM}}(T)$ exhibit an Arrhenius-like dependence on the temperature as discussed in the main text. The exponential

fit functions approximating the temperature dependent mean lifetimes $\overline{\tau}_{\mathrm{Sk}}(T, B)$ and $\overline{\tau}_{\mathrm{FM}}(T, B)$ at a specific field ($B = \mathrm{const.}$) intersect in $(T_{\mathrm{c}}, \overline{\tau}_{\mathrm{c}})$. This is shown exemplarily in the inset of Supplementary Figure 3 for the magnetic field $\mu B = 0.093\,J$. The point of intersection $(T_{\mathrm{c}}, \overline{\tau}_{\mathrm{c}})$ at which $\overline{\tau}_{\mathrm{Sk}} = \overline{\tau}_{\mathrm{FM}} = \overline{\tau}_{\mathrm{c}}$ is marked by a black circle and can be found again in the graph of the main panel. The other data points in the main panel were found in the same way by the intersections of $\overline{\tau}_{\mathrm{Sk}}(T)$ and $\overline{\tau}_{\mathrm{FM}}(T)$ at other magnetic fields. This procedure is possible because the point of intersection depends on the magnetic field, i.e. $(T_{\mathrm{c}}(B), \overline{\tau}_{\mathrm{c}}(B))$. The numerical results are quantified by the experimental data for Pd/Fe/Ir(111) as discussed in the main text providing absolute energy and time scales for the MC simulation. With this correspondence, we can derive that the skyrmionic and ferromagnetic states are stable on the order of years at the critical field B_{c} for temperatures lower than $19\,\mathrm{K}$.

Supplementary References

1. Ezawa, M. Compact merons and skyrmions in thin chiral magnetic films. *Phys. Rev. B* **83**, 100408 (2011).

2. Ramirez, A. P., Hayashi, A., Cava, R. J., Siddharthan, R. & Shastry, B. S. Zero-point entropy in 'spin ice'. *Nature* **399**, 333–335 (1999).

3. McMichael, R. D., Shull, R. D., Swartzendruber, L. J. & Bennet, L. H. Magnetocaloric effect in superparamagnets. *J. Magn. Magn. Mater.* **138**, 29–33 (1992).

4. Eyring, H. The activated complex in chemical reactions. *J. Chem. Phys.* **3**, 107–115 (1935).

5. Laidler, K. J. & King, M. C. The development of transition-state theory. *J. Phys. Chem.* **87**, 2657–2664 (1983).

6. Kramers, H. A. Brownian motion in a field of force and the diffusion model of chemical reactions. *Physica* **VII**, 284–304 (1940).

7. Nowak, U., Chantrell, R. W. & Kennedy, E. C. Monte Carlo simulation with time step quantification in terms of Langevin dynamics. *Phys. Rev. Lett.* **84**, 163–166 (2000).
